# HORNET: Tools to find genes with causal evidence and their regulatory networks using eQTLs

**DOI:** 10.1101/2024.10.28.24316273

**Published:** 2024-10-31

**Authors:** Noah Lorincz-Comi, Yihe Yang, Jayakrishnan Ajayakumar, Makaela Mews, Valentina Bermudez, William Bush, Xiaofeng Zhu

## Abstract

**Motivation:**

Nearly two decades of genome-wide association studies (GWAS) have identify thousands of disease-associated genetic variants, but very few genes with evidence of causality. Recent methodological advances demonstrate that Mendelian Randomization (MR) using expression quantitative loci (eQTLs) as instrumental variables can detect potential causal genes. However, existing MR approaches are not well suited to handle the complexity of eQTL GWAS data structure and so they are subject to bias, inflation, and incorrect inference.

**Results:**

We present a whole-genome regulatory network analysis tool (HORNET), which is a comprehensive set of statistical and computational tools to perform genome-wide searches for causal genes using summary level GWAS data that is robust to biases from multiple sources. Applying HORNET to schizophrenia, we identified differential magnitudes of gene expression causality. Applying HORNET to schizophrenia, we identified differential magnitudes of gene expression causality across different brain tissues.

**Availability and Implementation:**

Freely available at https://github.com/noahlorinczcomi/HORNETor Mac, Windows, and Linux users.

**Contact:**

njl96@case.edu .

## Full Text Availability

The license terms selected by the author(s) for this preprint version do not permit archiving in PMC. The full text is available from the preprint server.

